# Low-energy electron beam has severe impact on seedling development compared to cold atmospheric pressure plasma

**DOI:** 10.1038/s41598-021-95767-0

**Published:** 2021-08-12

**Authors:** A. Waskow, D. Butscher, G. Oberbossel, D. Klöti, P. Rudolf von Rohr, A. Büttner-Mainik, D. Drissner, M. Schuppler

**Affiliations:** 1 Institute of Food, Nutrition and Health, ETH Zurich, Schmelzbergstrasse 7, 8092 Zurich, Switzerland; 2 Institute of Process Engineering, ETH Zurich, Sonneggstrasse 3, 8092 Zurich, Switzerland; 3 Competence Division for Plants and Plant Products, Seed Quality, Agroscope, Reckenholzstrasse 191, 8046 Zurich, Switzerland; 4 Department of Life Sciences, Albstadt-Sigmaringen University, Anton-Günther-Strasse 51, 72488 Sigmaringen, Germany; 5 Present Address: Swiss Plasma Center, École Polytechnique Fédérale de Lausanne, Lausanne, Switzerland; 6 Present Address: BASF Personal Care and Nutrition GmbH, Illertissen, Germany

**Keywords:** Microbiology, Plant sciences, Materials science

## Abstract

Sprouts are germinated seeds that are often consumed due to their high nutritional content and health benefits. However, the conditions for germination strongly support the proliferation of present bacteria, including foodborne pathogens. Since sprouts are consumed raw or minimally processed, they are frequently linked to cases of food poisoning. Therefore, a seed decontamination method that provides efficient inactivation of microbial pathogens, while maintaining the germination capacity and quality of the seeds is in high demand. This study aimed to investigate and compare seed decontamination by cold atmospheric-pressure plasma and low-energy electron beam with respect to their impact on seed and seedling quality. The results show that both technologies provide great potential for inactivation of microorganisms on seeds, while cold plasma yielded a higher efficiency with 5 log units compared to a maximum of 3 log units after electron beam treatment. Both techniques accelerated seed germination, defined by the percentage of hypocotyl and leaf emergence at 3 days, with short plasma treatment (< 120 s) and all applied doses of electron beam treatment (8–60 kGy). However, even the lowest dose of electron beam treatment at 8 kGy in this study caused root abnormalities in seedlings, suggesting a detrimental effect on the seed tissue. Seeds treated with cold plasma had an eroded seed coat and increased seed wettability compared to electron beam treated seeds. However, these effects cannot explain the increase in the germination capacity of seeds as this was observed for both techniques. Future studies should focus on the investigation of the mechanisms causing accelerated seed germination and root abnormalities by characterizing the molecular and physiological impact of cold plasma and electron beam on seed tissue.

## Introduction

There has been an increase in foodborne illnesses due to the consumption of fresh produce in the United States and the European Union^1,2^. This has been attributed to the recent shift in consumers’ eating habits towards healthy, ready-to-eat and convenient foods that are minimally processed and minimally preserved^3,4^. Sprout consumption has also increased since sprouts are considered highly nutritious and provide numerous health benefits to consumers^5–8^.

Despite these benefits, sprout production requires warm, humid and nutrient-rich conditions which strongly support the proliferation of bacteria on seeds up to high levels^9–11^. The initial bacterial contamination on seeds can be traced back to different sources, such as seed production fields near animal rearing facilities, contaminated water, inadequate handling by workers, dirt and debris during harvesting or improper storage on the floor^12^. Since sprouts are usually consumed raw or minimally processed to limit nutrient loss, there is no adequate inactivation of microorganisms before consumption. Therefore, sprouts harbor a particular risk for the presence of foodborne pathogens, such as enterohemorrhagic *Escherichia coli* (EHEC), *Listeria monocytogenes* and *Salmonella* spp. with an increasing incidence of foodborne outbreaks during the last decade, like the outbreak of *E. coli* O104:H4 on fenugreek sprouts in 2011 with approximately 4,000 cases and 50 deaths in Germany^12–14^.

Since 2011, regulations have been tightened in the United States to maintain hygienic practices throughout the seed and sprout production and an antimicrobial treatment before seed germination is required to reduce the risk of foodborne illnesses. In the United States, 20 g/L calcium hypochlorite is applied on sprouts and achieves a 2.5 logarithmic (log) CFU/g reduction. However, its use is banned in some European countries due to its negative effect on human health and the environment^15–17^. Therefore, there is certainly a need for alternative sprout decontamination methods and several are under development, as discussed and reviewed in depth by Sikin et al.^17^. These include organic acids, electrolyzed water, ozone, bacteriophages, bacteriocins, irradiation, UV light, and high pressure. However, the authors concluded that these methods do not provide an effective antimicrobial treatment while maintaining germination capacity, nutritional and organoleptic quality with little to no negative impact on human health and the environment, which is also stressed by a more recent review^18^.

In recent years, novel technologies have emerged which seem to be promising for seed decontamination because they are safe, fast, and economical. Besides indirect treatment techniques, like plasma-activated water, which shows high efficacy for seed processing, direct treatment techniques, like cold atmospheric-pressure plasma (CAP) and low-energy electron beam (LEEB) also have great potential^19–22^. Plasma is a fourth state of matter and comprised of a partially or fully ionized gas which consists of UV photons, free electrons, negatively and positively charged ions, free radicals and neutral or excited atoms and molecules^23,24^. Depending on the operating gas, a diversity of reactive species is generated, such as reactive oxygen species (ROS) and nitrogen species (RNS)^25^. Under humid conditions, H_3_O^+^ and OH^−^ ions, OH radicals or hydrogen peroxide (H_2_O_2_) can be generated. Plasma is subdivided into thermal and non-thermal plasma. In particular, non-thermal plasma represents an attractive alternative treatment for thermo-labile materials like food. It consists of high-energy electrons and low-energy heavy particles and thus, the gas remains at a low temperature^23–25^. Non-thermal plasma can be ignited under low and atmospheric pressure. However, atmospheric-pressure plasma systems are preferred by industry due to lower costs, more applicability and short treatment times^26^. Low-energy electron beam (LEEB) processing is beta radiation consisting of a beam of accelerated electrons. It is a form of ionizing radiation applied in the range of a few to approx. 300 kGy. The accelerated electrons can remove electrons from atoms or molecules to produce ions^27^. Electron beam irradiation, therefore, requires a cathode in a vacuum environment to produce electrons that are accelerated close to the speed of light. In contrast to gamma irradiation, LEEB irradiation has a low penetration depth and works without a radioactive source such as Cobalt-60 or Cesium-137, which makes it more attractive for potential food applications. The application of CAP or LEEB as non-thermal techniques for seed decontamination and their effect on seed germination and seedling growth is discussed in several reviews^28–32^. However, it seems that complete eradication of microorganisms while maintaining the full germination capacity of seeds cannot be achieved by cold atmospheric-pressure plasma or low-energy electron beam, because improved antimicrobial efficiency due to an increase in treatment duration or input power usually results in a more severe impact on the germination capacity and viability of seeds^30,33–35^.

In this study, cold atmospheric-pressure plasma generated by a diffuse coplanar surface barrier discharge (DCSBD) and low-energy electron beam were applied for the inactivation of high levels of *E. coli* on the same batch of lentil seeds. Through the treatment of identical biological substrates in this study, we compared the efficiency of the techniques for the inactivation of microorganisms on the seed surface. Another major focus of the study was the investigation of the impact of CAP and LEEB treatment on the germination capacity and seedling development, to determine whether these techniques may cause deviations from expected plant development after seed treatment.

## Results

### Inactivation of *E. coli* on lentil seeds

**Figure 1:**
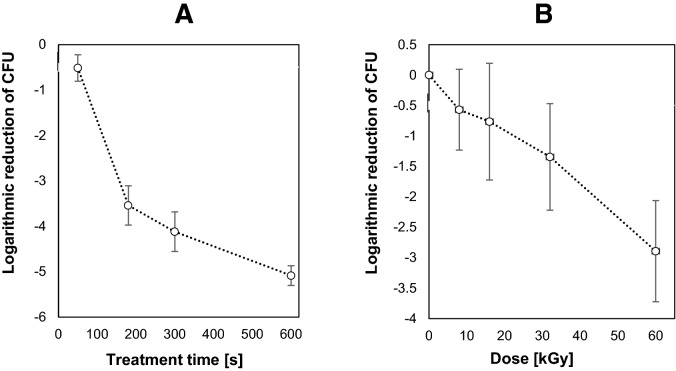
Inactivation kinetics for artificially inoculated *E. coli* on lentil seeds as a function of CAP treatment time (**A**) and inactivation kinetics for artificially inoculated *E. coli* on lentil seeds as a function of LEEB radiation dose (**B**). Error bars express standard deviation of the mean of triplicates for each data point.

The decontamination efficiency of CAP and LEEB was determined by exposing artificially inoculated lentil seeds to the cold atmospheric-pressure plasma generated on a diffuse coplanar surface barrier discharge (DCSBD) for 50 s, 180 s, 300 s and 600 s. The resulting log reductions were 0.52, 3.54, 4.12, and 5.09 log units, respectively, as shown in Fig. 1. For LEEB decontamination, artificially inoculated lentil seeds were exposed to radiation doses of 8 kGy, 16 kGy, 32 kGy, and 60 kGy, resulting in a log reduction of 0.57, 0.76, 1.34, and 2.89 log units, respectively. The aim of this experiment was to select the parameters that could attain the highest log reduction, which was approx. 5 log units after 10 min CAP treatment compared to approx. 3 log units achieved by LEEB at 60 kGy.

### Impact of treatment on seed quality

**Figure 2:**
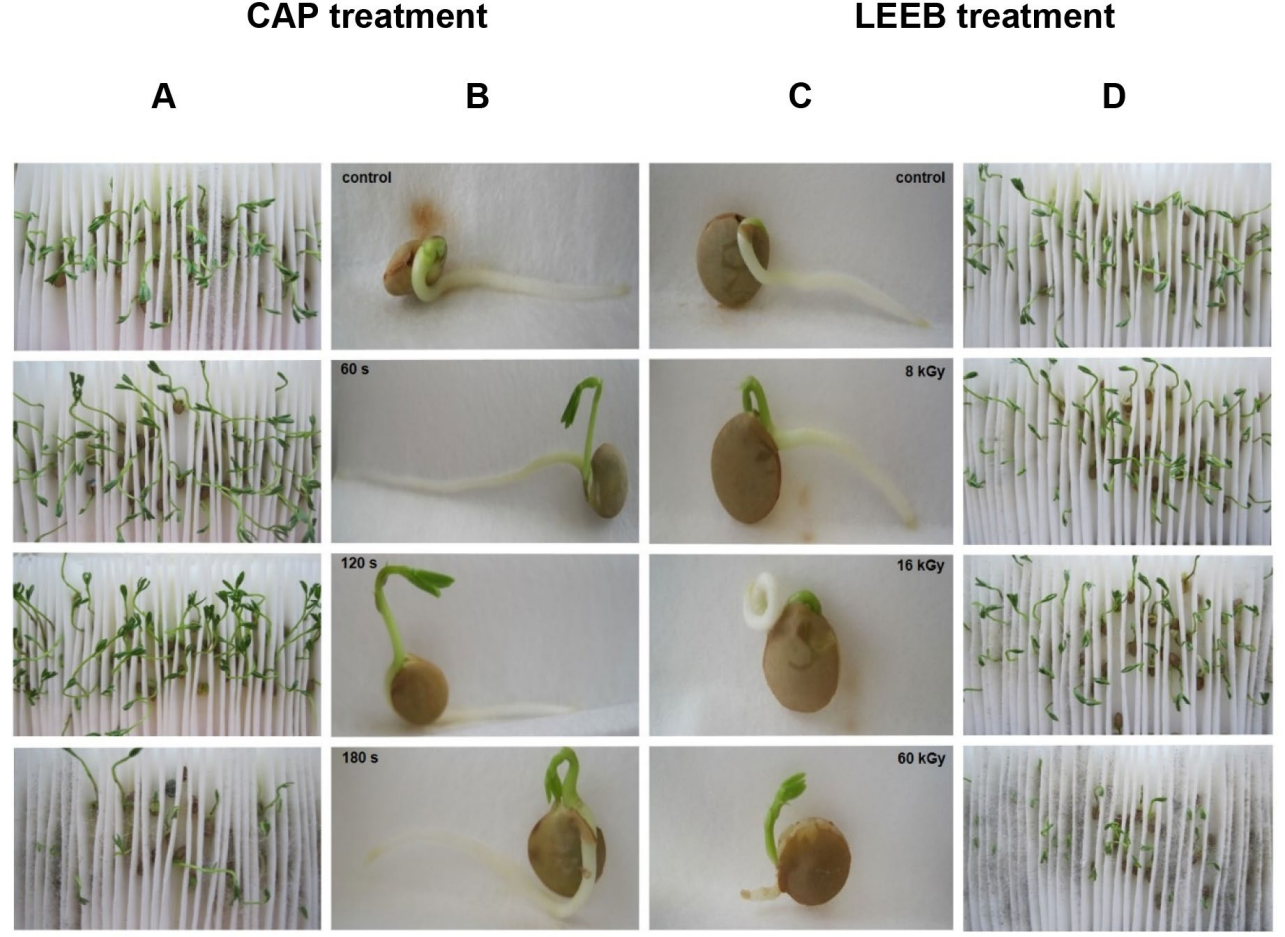
Morphology of seedlings resulting from CAP and LEEB treated lentil seeds. Qualitative assessment of 7-day-old (**A**) and 4-day-old (**B**) untreated and CAP treated lentil seeds with increasing CAP exposure time. Qualitative assessment of 4-day-old (**C**) and 7-day-old (**D**) untreated and LEEB treated seeds. Root abnormalities were observed for 8 kGy treatment and higher doses. Curling and shortening of roots was visible in seeds starting from 16 kGy treatment.

**Figure 3:**
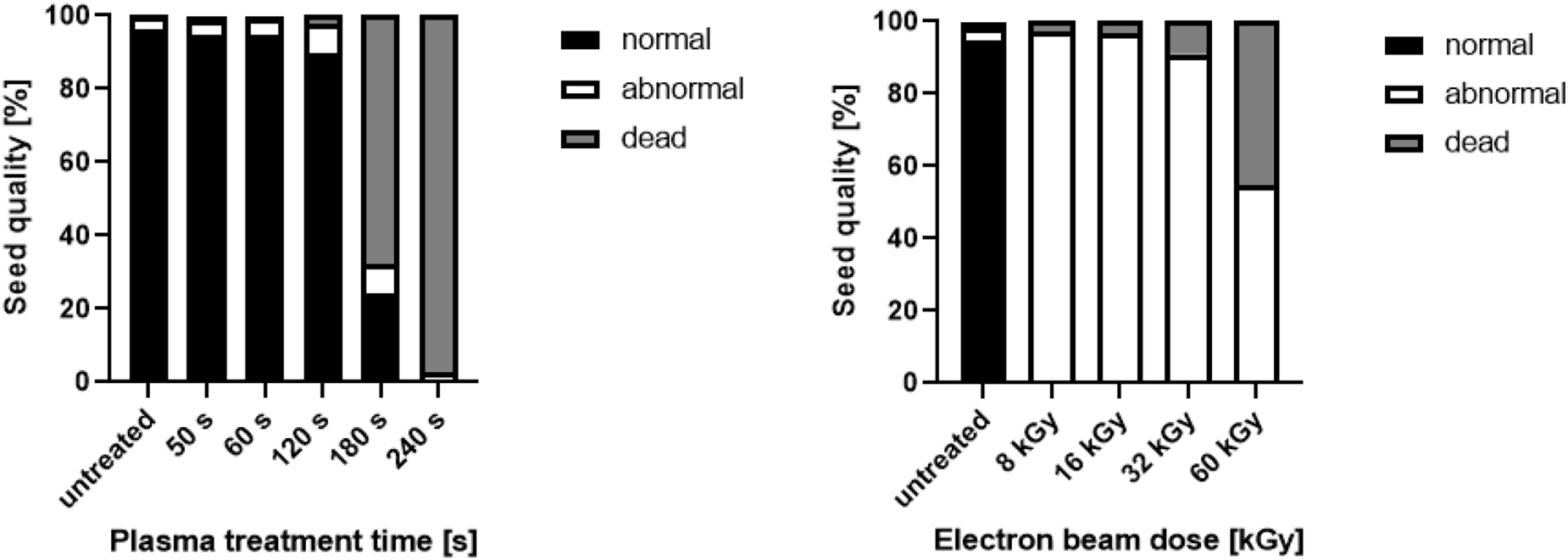
Quantitative assessment of 10-day-old seeds treated by CAP or LEEB. The percentage of seeds that resulted in normal, abnormal or dead seeds is indicated for increasing CAP exposure times (left) or LEEB doses (right).

A high germination capacity of seeds is of utmost importance for their use in sprout production. Therefore, the germination capacity of seeds was determined after different treatment regimes. Figure 2A,B indicates that the germination capacity of lentil seeds was unaffected up to 120 s exposure to CAP. However, prolonged treatment resulted in a high number of seeds which did not germinate, classified as dead seeds (Fig. 3). In comparison, LEEB treatment did not only negatively affect the germination capacity after high dose treatment (> 32 kGy), but resulted in a high percentage of seeds showing root abnormalities, such as shortened root length, root infection, or curly roots, already after mild treatment (Fig. 2C,D). Due to this observation seeds treated by LEEB, independent of the dose applied, were categorized as abnormal (Fig. 3).

In addition to the germination capacity, the vitality of treated seeds was investigated by performing a tetrazolium assay. Tetrazolium enters both, living and dead cells but only living cells catalyze the formation of formazan, which stains viable tissue red while dead tissue remains unstained. Consequently, viable seeds containing intact tissue show a red color, while damaged tissue in dead seeds has a white to yellow color. The extent and location of damaged and thus unstained tissue provides information about whether the seeds would have developed into normal or abnormal seedlings. A tetrazolium assay was performed on 15 seeds for each treatment regime.

The vitality of the seeds was negatively affected by increasing CAP exposure time. Up to 120 s CAP treatment did not negatively affect the seed tissue, while after 180 s CAP exposure time, two of 15 seeds revealed severe damage resulting in unstained tissue. After 240 s exposure time, 7 of 15 seeds were dead (data not shown).

After LEEB treatment of 8 kGy, only one dead seed was observed, which was comparable to the untreated control. Although the rest of seeds were classified as viable in the tetrazolium assay, all LEEB (8 kGy) treated seeds developed into abnormal seedlings in independent germination assays. Higher doses of up to 60 kGy resulted in approx. 50% dead seeds (Fig. 3). Furthermore, seed softening was observed with 60 kGy LEEB treatment. The high proportion of abnormal seedlings and dead seeds recorded in independent germination tests confirmed the findings from the tetrazolium assays.

### Acceleration in seedling development

**Figure 4:**
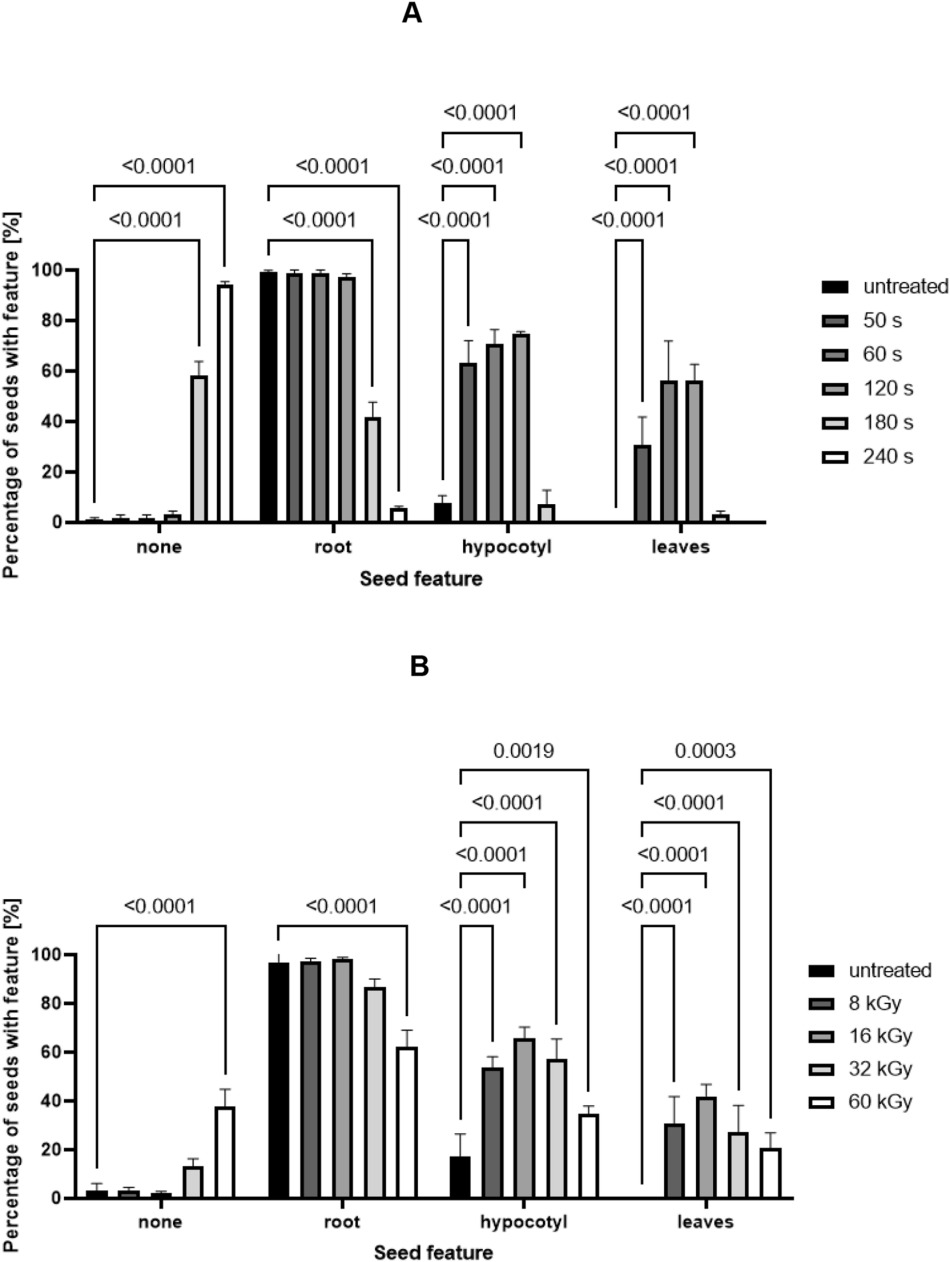
Quantitative assessment of seedlings grown from CAP and LEEB treated lentil seeds. (**A**) Quantitative assessment of 3-day-old seedlings from untreated and CAP treated seeds revealed accelerated seed germination compared to the untreated controls. Starting from 180 s, the germination capacity decreased and culminated in a complete lack of germination after 240 s treatment. (**B**) Quantitative assessment of 3-day-old seedlings grown from untreated and LEEB treated seeds revealed accelerated germination compared to the control, which is indicated by a higher percentage of seeds with hypocotyl and leaves. Starting from 32 kGy, the percentage of dormant or dead seeds increased. Columns are means of three independent experiments (N = 3), each comprising the analysis of 66 seeds. Significance of differences is indicated by the respective P-values.

**Figure 5:**
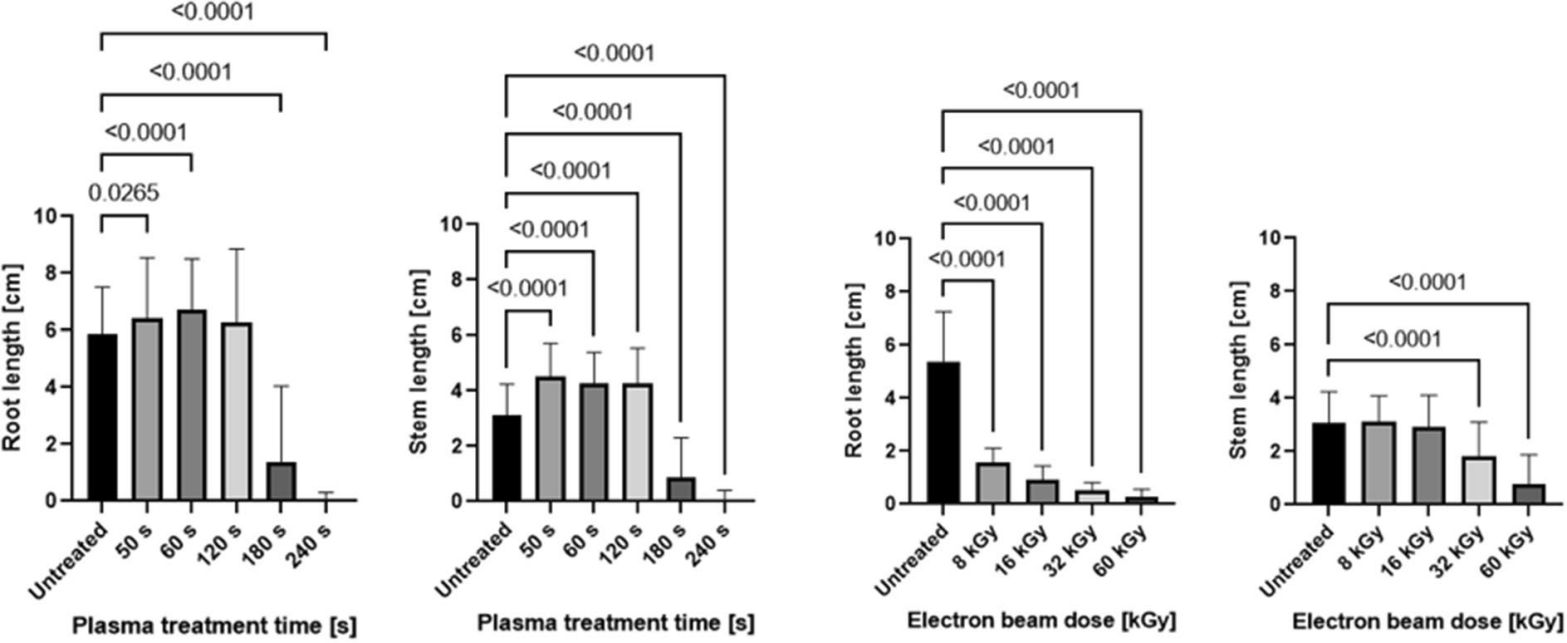
Assessment and comparison of root and stem length of 7-day-old seedlings grown from seeds treated by CAP or LEEB. The length of root and stem of seedlings was determined for increasing CAP exposure times (left) or LEEB doses (right). Columns are means of three independent experiments (N = 3), each comprising the analysis of 66 seeds. Significance of differences is indicated by the respective P-values.

CAP treatment (50 s, 60 s, and 120 s) and LEEB treatment of lentil seeds at all selected irradiation doses resulted in accelerated development of the shoot (hypocotyl) and the primary leaves (cotyledons) during germination (Fig. 4). The determination of root and stem length of seedlings after CAP and LEEB treatment of seeds revealed an increased root and stem (hypocotyl) length for 3-day-old seedlings grown from plasma-treated seeds (Fig. 5), where the increase in stem length was more pronounced than the elongation of the root. In contrast, LEEB treatment resulted generally in stunted, abnormal roots showing a curly morphology (Fig. 2C), while at higher dosage (32 and 60 kGy) the development of the stem was also impaired (Fig. 2D).

**Figure 6:**
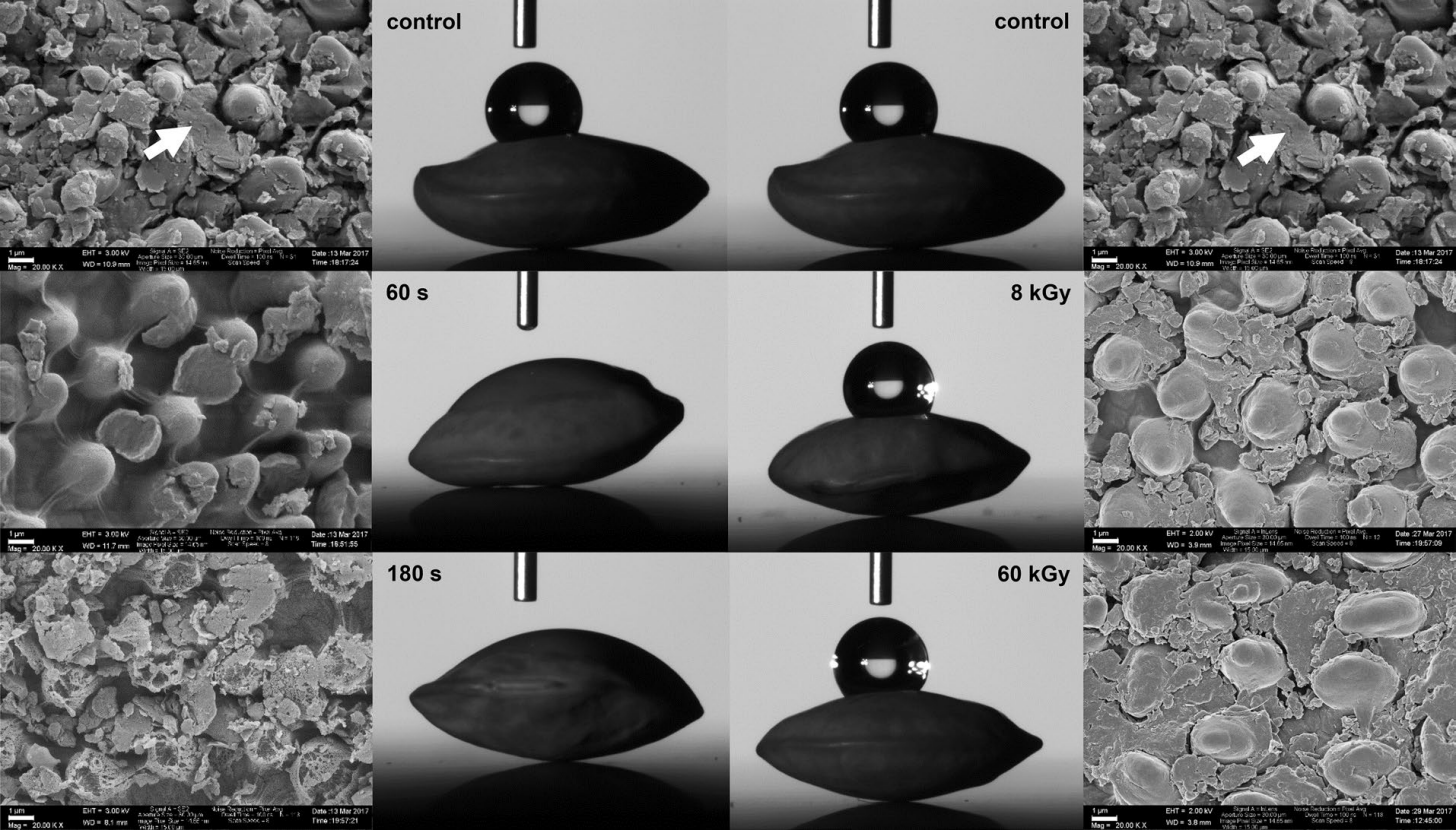
Scanning electron micrographs of the surface topology of CAP treated (left) or LEEB treated (right) lentil seeds. Scale bars represent 1 µm. Time of plasma exposure is indicated in the top left or right corner of the images. White arrows indicate embedded debris observed between the papillae of untreated seeds used as control. While seeds exposed to 60 s of CAP treatment revealed visible concave pits that were cleared from such debris, even high dose electron beam treatment did not remove the debris lodged between the protruding structures on the lentil seed surface. After 180 s CAP treatment, the seed surface revealed erosion of the protruding structures. The middle part of the figure displays the impact of CAP (left) or LEEB (right) treatment on the wettability of the lentil seeds shown with water droplet and contact angle measurements. Untreated control seeds showed a hydrophobic surface. While the surface of CAP treated seeds became hydrophilic, LEEB treated seeds maintained hydrophobic surface characteristics. SEM images provided by Stephan Handschin, CC by 4.0.

To characterize the mechanisms promoting the germination of seeds, the surface of treated and untreated seeds was analyzed using scanning electron microscopy (SEM) and wettability measurements. Figure 6 shows that untreated seeds had debris lodged between protruding structures (papillae) on the surface of seeds. These structures with debris are common for several lentil varieties and have already been described earlier^36^. Seeds exposed to CAP revealed visible concave pits that were cleared from such debris. After 180 s CAP treatment, the seed surface showed erosion of the protruding structures. In contrast, even high dose in LEEB treatment did not remove the debris lodged between the papillae on the lentil seed surface. Another observation, which is most probably linked to the altered microstructure on the seed coat, was related to the wettability of the surface of treated seeds. Figure 6 illustrates the different effects of CAP and LEEB treatment of seeds. While CAP exposure for 60 s and 180 s drastically increased the wettability of the seed surface relative to the untreated controls, LEEB treatment of lentil seeds did not influence the wettability, as the surface of LEEB treated seeds remained strongly hydrophobic.

## Discussion

The results from this study indicate that both, CAP and LEEB, are efficient technologies for reducing the microbial load on seeds. While CAP provides an advantage over LEEB in terms of the log reduction, LEEB treatment is advantageous because of the very short treatment times regardless of the dose (e.g. 3 log reduction in 10 s). A 3 log reduction was achieved for *E. coli* on lentil seeds after 180 s CAP treatment and even longer treatment times are needed to succeed in a maximum reduction of 5 log. In a previous study using the same DCSBD device, a maximum log reduction of 8.8 log units was achieved after 10 min CAP treatment for Gram-positive foodborne pathogens such as *Listeria monocytogenes* and *Staphylococcus aureus*^35^. The highest log reduction after 3 min CAP treatment was 5.2 log units obtained for Gram-negative *E*. *coli*, which is in accordance with the results from this study. In this context, it should be mentioned that the decontamination efficiency for CAP treatment depends on the moisture content of the lentils. It was previously shown that moisture content can trigger a change in the liquid chemistry^37^. Thus, it is an important parameter that may be optimized to increase the plasma decontamination efficiency. However, if the moisture content is too high, it may result in the quenching of plasma by decreasing the plasma intensity as the energy is redirected to the rotational and vibrational excitation of molecules instead of ionization^38^. Approximately, a 2% increase or decrease in moisture content can alter the decontamination efficiency of plasma by approximately 0.5 to 1 log units, respectively (data not shown). Only limited data are available on the influence of moisture on the efficiency of electron beam treatment, but it seems that moisture may also impact the decontamination efficiency of LEEB because energy may be redirected to the radiolysis of water^39,40^. The radiolysis of water may lead to the formation of endogenous reactive species but since they most likely remain inside the seeds, they do not interact with microorganisms on the seed surface. Furthermore, the concentration of reactive oxygen species and reactive nitrogen species may be lower with LEEB treatment, since the atmospheric oxygen is replaced with pure nitrogen gas prior to the treatment. This might be an explanation for the lower effectivity in seed decontamination compared to CAP.

Considering the impact of treatment on the germination capacity of seeds, the results obtained in this study are consistent with several other studies. When dosed appropriately, CAP treatment can maintain or even improve the overall germination capacity of various plant species such as alfalfa seeds or wheat seeds^32,35,41,42^. A study using the same DCSBD air plasma device like the one used in this study also confirmed the positive impact on the germination capacity of wheat seeds^43^. The authors showed an increase in germination rate with treatment times below 60 s and a decrease in the germination rate after prolonged plasma treatment, which is in accordance with our results and previous reports^44–46^. In particular, a dramatic increase in germination speed with short plasma treatment times between 15 s and 2 min was observed^46^.

Interestingly, the application of LEEB treatment in this study also resulted in accelerated germination of seeds. In the meantime, LEEB has been developed further and is offered as a standard seed treatment for wheat disinfection in biological farming^30^. The use of LEEB for treatment of wheat seeds revealed a positive impact on the germination capacity of treated seeds^47^. However, another previously published study reported that the germination capacity of mung beans, clover seeds and fenugreek seeds was not affected by electron beam doses of up to 12 kGy^48^.

Although there are observed positive effects of CAP and LEEB treatment on the germination of seeds, it is still unclear which mechanisms are behind the improved seed germination and viability^32^. Some authors attributed the increased germination speed to an improved wettability of the seeds and reported a noticeable change in water imbibition for air plasma treated lentils and wheat grains^45,46^. They explained that air plasma deposits oxygen and nitrogen containing groups on the outer layer of the seed tissue, which could modify the O_2_ and CO_2_ permeability and increase seed wettability. Another study that observed a positive effect of low-pressure plasma treatment on sunflower seed germination as well as on sprout length and dry weight argued that the significant increase in wettability is caused by RONS, which oxidize lipids present in seed coat leading to a hydrophilic property which promotes seed water uptake and thus seed germination^49^. However, the removal of waxy structures from the surface of seeds by plasma etching and the interaction of reactive chemical species in plasma are also mentioned as a potential reason for changing the chemistry of the seed surface, which may improve surface wettability and water uptake^32^. As discussed in a recent review, analysis of the surface of quinoa seeds by XPS (X-ray photoelectron spectroscopy) revealed that the seed surface was largely affected by non-thermal plasma and attenuated total reflection-Fourier-transform infrared spectroscopy (ATR-FTIR) measurements indicated changes in the chemical groups of plasma-treated seeds, which point to a decrease in the lipid group and an increase in polar groups containing oxygen and nitrogen^32^. In contrast to CAP, the wettability of seeds treated with LEEB remained unaffected, which may be explained by the very low concentration (< 200 ppm) of oxygen present in the treatment chamber of the electron beam system. Hitherto, no further data exist on the influence of electron beam treatment on the wettability of seeds. Only one study could be recovered that studied the impact of electron beam on tooth enamel and the authors observed an increase in hydrophobicity^50^. Although tooth enamel and the surface material of seeds are clearly different substrates, this study may provide indirect evidence for the lack of an increase in hydrophilicity after LEEB treatment of lentil seeds.

Another potential explanation for the positive impact of short plasma exposure times (< 120 s) on the germination capacity of seeds might be scarification of the seed surface, which was observed after CAP treatment of seeds in this study (Fig. 6). This process may lead to mild erosion and clearing of debris on the seed coat, thus allowing for improved water and gas transport. However, SEM analysis of the seed surface topology of LEEB treated lentil seeds revealed no such effect. Therefore, it is unlikely that this process is responsible for the accelerated germination observed after CAP and LEEB treatment of seeds.

In conclusion, the results from this study suggest that the observed accelerated germination after both CAP and LEEB treatment is not due to an increased wettability or scarification of the seed surface. Thus, another explanation might be the generation of ROS and/or RNS which is the case for both technologies. The chemical composition of the gaseous products of LTP plasma was already investigated by FTIR spectroscopy in several studies^51–53^. Their results showed that gas of low-temperature plasma generated in air is typically composed of ROS and RNS such as NO_2_, N_2_O, NO, O_3_, HNO_3_, HNO_2_, and CO_2_. Consequently, air micro-plasma was most efficient in improving seed germination rate and seedling growth of mung beans^54^. Also, the role of reactive oxygen species in regulating dormancy of *Arabidopsis* seeds has been reported and non-dormant seeds revealed higher ROS contents with superoxide and hydrogen peroxide accumulating near the radicle^55^. Nitric oxide, hydroxyl radicals and superoxide radicals, accumulate during germination and provide a positive signal in seed dormancy release^56^. Therefore, the concept of the “oxidative window for germination” described previously by Bailly et al. might be the key to understanding the effects occurring with CAP treatment of various intensities^57^.

Recent studies showed that the level of superoxide anion and nitric oxide were significantly higher in seeds after short (up to 180 s) plasma treatments, while the level of hydrogen peroxide was remarkably increased in long-time (> 5 min) plasma-treated seeds^58^. Moreover, an interplay is suggested between hydrogen peroxide and the plant hormones gibberellic acid (GA) and abscisic acid (ABA), which are important in regulating seed germination and dormancy. This assumption is supported by a study which showed that an interaction between ROS and the plant hormone GA leads to signaling that causes increased stem length^59^. This would explain the elongated stems observed after plasma treatment (Fig. 5), which was also reported previously, except for soybean sprouts^60^. Furthermore, a study on the effect of low-temperature plasma on seed growth and metabolism of endogenous phytohormones in pea, where a noticeable increase in germination rate as well as in root and shoot length was observed, reported an increased biosynthesis of auxin and cytokinins as well as their catabolites and conjugates^61^.

In this study, root and stem elongation were observed for CAP treated but not for LEEB treated seeds. In contrast, LEEB treatment resulted in the occurrence of root abnormalities such as stunted, curled roots and the tetrazolium test confirmed damaged root tip tissue (data not shown). It was previously described that the UV photons in plasma are restricted to the upper one micrometer layer and it is therefore unlikely that they cause damage, whereas electron beam treatment is able to cause damage to cells in deeper tissue due to the higher penetration depth of electrons^62,63^. Since the root is located close to the edge of the seed, it would be the most susceptible structure, whereas the stem is better shielded and hidden by the endosperm. The impaired development of the roots of seedlings grown from the seeds treated by LEEB, even at low doses, suggests that LEEB treatment may trigger so far unknown processes in the seed tissue. Given the observed relationship between root curling, and the involvement of auxin transport, Santner et al. speculate that alterations in auxin transport may account for this distinctive phenotype^64^. It is known that the root tip can synthesize auxin and that the asymmetric localization of auxin efflux carriers in the plasma membrane determines the polarity of auxin transport^65,66^. Upon environmental stimulation, these carriers may relocate and subsequently alter the overall growth response of the organ^67^. Therefore, the observed curling of the roots after LEEB treatment of seeds in this study suggests that LEEB may affect auxin function in the root tip tissue.

Since electron beam is known to inactivate microorganisms by direct and indirect DNA damage^68^, it is conceivable that the root abnormalities are due to DNA damage of the root tissue. However, this would need to be verified by further research. It might be possible to avoid the root abnormalities using lower doses of electron beam treatment. Nonetheless, even doses as low as 1.2 kGy were reported to cause a high percentage of abnormal seedlings^47^. Interestingly, a study that investigated the effect of gamma radiation on seed germination and seedling growth of *Lathyrus chrysanthus* Boiss. under in vitro conditions did not observe any similar effects^69^. The results showed that irradiated seeds revealed an increased seed germination percentage and increased seedling and root lengths. However, higher doses resulted in a significant decrease in all parameters.

Plasma treatment might stimulate the plant immune system to produce ROS^55,56^. In contrast, electron beam may produce mainly endogenous reactive species, which would explain the lower log reduction, since these reactive species remain inside the seed tissue and cannot interact with microorganisms on the surface. On the other hand, a high concentration of reactive species in the tissue could also be an explanation for the observed root tip tissue damage, as it is known that endogenous ROS accumulate at the radicle when seeds are released from dormancy.

## Conclusion

This study provides the first comparison of non-thermal plasma and low-energy electron beam treatment for seed decontamination of seeds from the same batch. In addition to the inactivation kinetics for high numbers of *E. coli* on the surface of lentil seeds, a major focus of the study was to investigate and compare the impact of the different treatment techniques on the quality attributes of the resulting sprouts, including the germination properties of the seeds and the morphology of seedlings. CAP treatment appeared to be highly efficient in the reduction of the microbial loads on the seed surface and was slightly advantageous over electron beam treatment in terms of the maximal reduction efficiency. Moreover, CAP treatment was superior to LEEB with regards to the germination capacity. However, it is still unclear which mechanisms are behind the improved seed germination and viability. The findings from this study suggest that generation of reactive species may trigger the observed accelerated germination rather than scarification or improved wettability, since both CAP and LEEB promoted the germination of seeds. While CAP treatment maintained the typical seedling morphology at least with short treatment times, even low doses of LEEB treatment resulted in an impaired root development in seedlings, suggesting that LEEB triggers so far unknown processes in the seed tissue that could interfere with auxin guided root development. In conclusion, both techniques show great potential for seed decontamination, but their application is largely hampered by the detrimental effect on seed germination and seedling development under intense treatment conditions, which would be necessary to achieve high microbial reduction rates. The results from this study show that further research will be necessary in order to better understand the mode and mechanisms of action that affect seed germination and seedling development after CAP or LEEB treatment.

## Methods

### Sample preparation

*E. coli* ATCC 8739 was selected as a Gram-negative model organism to compare CAP and LEEB decontamination efficiency on lentil seeds. Sample preparation was performed as described elsewhere^35^. In brief, an overnight culture of *E*. *coli* was grown in 10 mL of LB broth (Merck Millipore, USA) and incubated at 37 °C. The cell number of the overnight culture was estimated by its optical density at 600 nm, where an optical density of 1 corresponded to a concentration of approx. 10^9^ CFU/mL. In a laminar flow bench, 1 g of lentils (Bigler Samen AG, Steffisburg, Switzerland) was placed in a Falcon tube, drizzled with 1 mL of the overnight culture (approx. 10^10^ CFU/mL), thoroughly mixed for 5 min and air dried in sterile petri dishes for 4 h.

### Cold atmospheric-pressure plasma treatment of lentil seeds

Seeds were treated using an atmospheric pressure diffuse coplanar surface barrier discharge (DCSBD) developed by Robust Plasma Systems (RPS600; Roplass s.r.o., Brno, Czechia) that has already been used and characterized in previous studies^22,70–73^. Detailed information about the DCSBD system used in this study is provided by Cernák et al., who describe the basic properties, mechanism as well as applications of ambient air DCSBD^72^. An overview about the DCSBD parameters applied in this study is given in Table 1.

**Table 1:** Characterization of DCSBD parameters. Values recorded as average of two measurements.

Intensity (%)	Power (W)	Voltage (kV)	Current (mA)	Frequency (kHz)
0	535.5	6.52	534	16.15
50	693.5	6.985	608.5	14.55
75	776	7.94	664	13.84
100	858.5	9.25	759	13.335

It contains strip-line structure electrodes embedded in a 96 mm × 230 mm alumina ceramic dielectric plate and ignites 80 mm × 200 mm plasma with 0.3 mm thickness using ambient air as the operating gas. The generation of plasma was performed with a sinusoidal power supply (9.25 kV, 13.33 kHz) and measured using a voltage probe (P6015, Tektronix), a current probe (Model 2877, Pearson Electronics) and monitored with 600 MHz oscilloscope (WaveRunner 64 Xi, LeCroy). The plasma temperature was approximated with a fiber-optic suspended above the ceramic plate using a sensor measuring the temperature dependent band gap shift of a gallium arsenide crystal (TS2/3, Polytec) connected to an appendant spectrometer (FOTEMP-Multichannel). Relative humidity was measured with a Series 1100 Hygrometer (Rotronic, Switzerland). Ozone is inherently produced by low temperature plasma ignited in air due to the dissociation of oxygen molecules contained by the air and can be detected from its UV absorption around 254 nm. Therefore, integration of optical emission over the 200–1000 nm wavelength range was performed using an optical fiber (QP400-3-SR-BX, Ocean Optics) connected to a spectrometer (USB2000 + XR1-ES). More details about the production of reactive species and their concentrations is provided by a recent publication that used optical emission spectroscopy to determine ozone, nitrous oxide, and nitrogen dioxide generated by the coplanar dielectric barrier discharge system^73^.

Prior to plasma decontamination experiments, the dielectric ceramic plate was disinfected with ethanol (70% v/v) to avoid cross contamination and evaporation of ethanol residues which otherwise may interfere with the treatment. Untreated, inoculated seeds served as reference for the determination of the reduction rate after the treatment of inoculated samples. For CAP treatment, 1 g of inoculated lentil seeds were distributed on the surface of the dielectric ceramic plate. Plasma was powered up to 100% intensity and powered down according to the time intervals for the respective treatment time (50–600 s). To ensure homogeneous treatment, lentils were treated on both sides by flipping them manually using sterile forceps after half of the treatment time. After the treatment, seeds were transferred into sterile Falcon tubes for subsequent determination of bacterial counts.

### Low-energy electron beam treatment of lentil seeds

The EBLab-200 system (Comet Group, Flamatt, Switzerland) was used for LEEB treatment of lentil seeds. Seed samples were treated in a tray with a dimension of 216 mm × 279 mm × 50 mm that moved through the irradiation chamber at a speed of 30 m/min. The sample surface had a distance of 20 mm from the electron-emitting lamp and the system was cooled by a minimum flow rate of 3 L/min cooling water. Treatment of both sides of the lentils was performed using radiation doses from 4 to 30 kGy with corresponding current of 1.088–8.163 mA. All treatments were performed at acceleration voltages of 180 kV, using a density of 1.2 g/cm^3^ (seeds). Prior to treatment, the oxygen concentration was set below 200 ppm by flushing with nitrogen (gas grade purity 6.0). After the treatment, seeds were transferred into sterile Falcon tubes for determination of bacterial counts. A detailed description of the technical specifications of the EBLab-200 system is available from the operating instructions provided by the Comet Group^74^. The parameters used for treatment of lentil seeds by cold atmospheric pressure plasma and low energy electron beam are compared in Table 2.

**Table 2:** Comparison of cold atmospheric pressure plasma (CAP) and low energy electron beam (LEEB) parameters used for treatment of lentil seeds in this study.

Parameters	CAP	LEEB
Treatment substrate	Lentils	Lentils
Treatment time	0–10 min	100 ms
Treatment area	80 mm × 200 mm	216 mm × 279 mm
Power/energy	858.5 W	Acceleration voltage of 180 kV
Gas type	Air	Nitrogen
Chemistry	[73, 74]	[39, 40]

Furthermore, a detailed comparison of cold atmospheric pressure plasma and low energy electron beam as alternative nonthermal decontamination technologies is provided by a recent review from Hertwig et al.^30^.

### Determination of bacterial counts

As previously described in Butscher et al., the inactivation rate for bacteria was determined by adding 9 mL of PBS (phosphate buffered saline, pH 7.4) to 1 g of lentils in a sterile blender bag (Huber) and homogenization for 3 min in a stomacher (AES laboratoire) prior to preparation of a decimal dilution series in PBS^37^. To determine the colony forming units (CFU) per gram of seeds for *E. coli*, standard plate count analysis was performed using an aliquot of 0.1 mL of the respective dilutions on chromogenic coliform agar (Biolife, Italy), incubated at 37 °C for 24 h and subsequent enumeration of purple colonies.

### Germination and viability testing of seeds

As a reference for seed quality, the germination capacity of seeds was determined according to the “International Rules for Seed Testing” specified by the International Seed Testing Association (ISTA). The assessment of seed germination was performed with untreated control seeds and treated seeds by the Agroscope, Seed Quality Laboratory, Zurich, Switzerland. In brief, seeds were placed in pairs into pleated paper (Ahlstrom-Munksjö), soaked in 40 mL of tap water and kept at a germination temperature of 20 °C at 85% humidity in a climate chamber (Kälte 3000, Switzerland) with an 8 h day with light (Lux = 4000) and 16 h night cycle^75^.

Seeds and seedlings were inspected and counted at the mid-count (7 days) and the final count (10 days). By day 7, germinating seeds were enumerated and classified as normal (healthy), or abnormal (unhealthy) according to International Seed Testing Association 2003, supplement 2011. In the case of complete absence of a root, the seeds were classified as dead. Non-germinated seeds were given time until day 10 to germinate before being classified as dead. For this analysis, a statistically significant sample size of 66 seeds was used and the analysis was performed in triplicates. The germination capacity was calculated using the formula below:

$$Germination \;capacity = 100\% \cdot \frac{{\sum {number \;of \;normal \;seedlings \;in \;all \;three \;replicates } }}{total \;number \;of \;seeds \;(198)}.$$

For the determination of germination rates, seed samples were enumerated as percentage of seeds with either the presence of root, hypocotyl and cotyledons (leaves), in other words, the stages of germination, and were enumerated on day 3, 4, and 7. The germination rate was calculated using the formula below:

$$Germination \;rate = 100\% \cdot \frac{{\sum {number \;of \;seed \;or \;seedlings \;with \;selected \;feature} }}{total\; number\; of\; seeds\; (198)}.$$

In order to specify the quality of the resulting seedlings, root length and shoot length was determined for all germinated seeds (66 seeds in triplicates) and averaged with their corresponding standard deviation. The root and stem length were determined for 7-day-old seedlings.

Furthermore, a tetrazolium assay was performed to determine the impact of CAP or LEEB treatment on the vitality of the seed tissue. The tetrazolium (2,3,5 triphenyl tetrazolium chloride) assay is a fast evaluation for seed viability^76^. All respiring tissues are capable of converting the colorless compound tetrazolium to a carmine red colored water-insoluble formazan by hydrogen transfer reaction catalyzed by the cellular dehydrogenases. Tetrazolium enters both living and dead cells but only living cells catalyze the formation of formazan. The non-diffusible formazan stains viable tissue red whereas absence of cellular respiration prevents formazan production. Thus, the tissue of dead seeds remains unstained. In the case of damaged seed tissue, the quantity and location of unstained tissue is relevant to classify the seeds into groups: those which would have yielded normal, healthy seedlings (viable), and those which would have germinated but developed into seedlings with severe abnormalities (non-viable). In conclusion, three categories of seeds were distinguished using the tetrazolium assay: (i) viable, (ii) non-viable seeds and (iii) dead seeds. This classification was applied according to the International Seed Testing Association (ISTA) Working Sheets on Tetrazolium Testing (1st edition 2003, with supplements 2011; agricultural, vegetable and horticultural species, Volume I). For each treatment regime (CAP and LEEB), 15 seeds were soaked in water for 18 h at room temperature. Next, the distal end of the cotyledons was cut off transversally and soaked in 1% tetrazolium solution for 6 h at 30 °C. After removal of the seed coat, it was determined whether and to which extent the seeds showed healthy tissue (red color) or damaged tissue (white to yellow color).

### Statistics

Differences between two groups were assessed using ordinary one-way ANOVA. Differences between more than two groups were assessed by using two-way ANOVA. GraphPad Prism 9 (GraphPad Software, Inc.) was used for statistical analyses. All P-values < 0.05 were considered to be significant and given directly in the plot. Only significant P-values are shown in the graphs.

### Determination of surface wettability

For determination of the surface wettability of treated and untreated seeds, contact angle measurements were performed using a Drop Shape Analyzer DSA25 (Krüss GmbH, Germany) with Software ADVANCE for intelligent image evaluation algorithm. For each seed, the contact angle was measured between the surface of the seed and liquid by placing 1 µL of distilled water at 0.1 µL/s at 26.0 °C. This was performed on three seeds for each condition.

### Scanning electron microscopy

Scanning electron microscopy (SEM) images were taken for untreated and treated lentil seeds to study their surface topology. Seeds were fixed onto aluminum specimen stubs with conductive carbon glue and sputter coated with 5 nm of platinum/palladium (CCU-010; Safematic). Samples were analyzed with the inlens and SE2 detector of a Leo 1530 (Zeiss) at an acceleration voltage of 2 kV and a working distance between 4 and 7 mm^37^.
